# Unraveling the Potential Pathogenic Role of Squalene Synthase (SQS) in Lung Cancer Using Enzyme Inhibitors as Molecular Tools

**DOI:** 10.1021/acsmedchemlett.5c00458

**Published:** 2025-09-11

**Authors:** Theodora Katavati, Filippos P. Chatzipieris, Christiana Magkrioti, Elli-Anna Stylianaki, Emmanouil Aerakis, Sofia Grammenoudi, Maria Tsoumakidou, Vassilis Aidinis, Alexios N. Matralis, Angeliki P. Kourounakis

**Affiliations:** †Institute for Bioinnovation and ‡Institute for Fundamental Biomedical Research, 54573 Biomedical Sciences Research Center “Alexander Fleming”, 16672 Athens, Greece; § Department of Medicinal Chemistry, School of Pharmacy, University of Athens, 15771 Athens, Greece

**Keywords:** Squalene Synthase (SQS), inhibitors, cholesterol, lung cancer

## Abstract

Squalene synthase (SQS) is a key enzyme in the mevalonate pathway, catalyzing the first committed step in cholesterol biosynthesis. Several small-molecule SQS inhibitors have been developed thus far, initially aiming at treating hypercholesterolemia. Although their development for cardiovascular diseases was limited after clinical trials compared with statins, the potential of SQS as an anticancer target has recently gained renewed attention. Nevertheless, the evaluation of SQS inhibitors as anticancer agents has been rudimentary thus far. Herein, we attempted to provide some clear evidence of the potential of this enzyme as a “druggable” target in lung cancer. Accordingly, two chemically diverse SQS inhibitors were used as pharmacological tools and tested in different cancer-cell-based assays, providing some groundwork for their mechanistic underpinning in cancer cells. Lastly, *in vivo* studies corroborated the potent effect of SQS inhibition on lung inflammation and tumor burden, rendering SQS as a viable and promising drug target against lung cancer.

## Graphical abstract

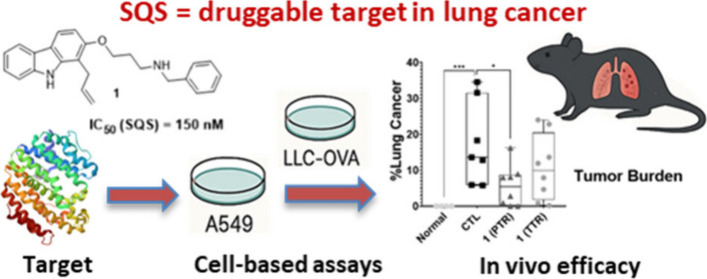

Squalene synthase (SQS) is a key enzyme in the mevalonate pathway, where it catalyzes the first committed step in cholesterol biosynthesis by converting farnesyl pyrophosphate (FPP) into squalene. This reaction represents a critical branching point, diverting FPP from other isoprenoid pathways toward sterol synthesis, thus controlling the production of cholesterol but also influencing the biosynthesis of other sterol-derived molecules essential for cellular function. As cholesterol and its derivatives play vital roles in cell membrane integrity, signal transduction, and lipid raft formation, the regulation of SQS activity is tightly controlled in normal physiology.^1–3^

Cancer cells often exhibit altered lipid metabolism to support their rapid proliferation and survival, and the upregulation of cholesterol biosynthesis has been recognized as a hallmark of tumorigenesis.^4,5^ Especially, the overproduction of cholesterol and related sterols contributes to the formation of lipid rafts, which serve as platforms for oncogenic signaling pathways, such as epidermal growth factor receptor (EGFR), Phosphoinositide 3-kinase (PI3K)/Akt, and rat sarcoma (Ras)/Mitogen-activated protein kinase (MAPK)—all frequently dysregulated in lung cancer and contribute to aggressive tumor behavior.^6,7^ Emerging evidence suggests that dysregulation of SQS is implicated in various cancer types. In specific, elevated expression of SQS has been observed in several malignancies, particularly in non-small cell lung cancer (NSCLC),^8,9^ but also in prostate^10^ and colon cancer,^11^ as well as in glioblastoma,^12^ and is often associated with enhanced tumor growth, poor prognosis, and therapy resistance. The main mechanism through which SQS is involved in cancer progression is by enhancing cholesterol biosynthesis, which is crucial for lipid raft formation (which in turn is important in signaling),^13^ steroid hormone synthesis, membrane fluidity, and cell division, thereby supporting oncogenic cascades (e.g., EGFR, PI3K/Akt, and Ras/MAPK). Of note, it was found recently that SQS plays also a pivotal role in promoting the invasion/migration of lung cancer cells via either the osteopontin/extracellular-signal-regulated kinase (ERK) pathway^14^ or the nuclear factor kappa-light-chain-enhancer of activated B cells (NF-kB)/matrix metallopeptidase 1 (MMP1) axis by facilitating tumor necrosis factor receptor 1 (TNFR1) enrichment into lipid rafts.^8^ The latter has rendered SQS a representative prognostic biomarker for lung cancer metastasis. Moreover, squalene accumulation itself may have cytoprotective effects, which can be reversed by SQS inhibition, thereby promoting oxidative stress and ferroptotic cell death in cancer cells.^3^ As a result, the dual effect of cholesterol depletion and pro-oxidant stress bestows SQS inhibitors a multifaceted anticancer mechanism.^15^

Several small-molecule SQS inhibitors have been developed thus far, initially aimed at treating hypercholesterolemia, including zaragozic acids,^16^ lapaquistat acetate (TAK-475),^17^ YM-53601,^18^ and many others.^19–22^ These compounds function by binding to the active site of SQS, thereby blocking the conversion of FPP to squalene. Although their development for cardiovascular diseases was limited due to hepatotoxicity or inadequate efficacy compared to statins,^23^ their potential as anticancer agents has gained renewed attention. Preclinical studies have demonstrated that inhibiting SQS suppresses tumor cell proliferation, induces apoptosis, and enhances sensitivity to chemotherapeutic agents. For instance, YM-53601 was shown to reduce tumor growth in glioblastoma cancer models by decreasing intracellular cholesterol levels and disrupting lipid raft-associated signaling, particularly the PI3K/Akt and EGFR pathways.^12^ In NSCLC, SQS knockdown or pharmacological inhibition using zaragozic acid A led to impaired cancer cell viability and tumorigenic potential.^8,9^ Moreover, the recent identification of the first non-conventional small molecules that can act either as degraders or stabilizers of SQS will shed light to alternative pharmacological concepts about the tractability of this enzyme as a new therapeutic strategy for cancer.^24^

Despite their oncogenic potential, the evaluation of SQS inhibitors against different types of cancer remains highly underexplored and limited to only two molecules, the natural product zaragozic acid A,^8^ and the quinuclidine derivative YM-53601,^12^ while the clinical translation of SQS inhibitors as anticancer agents remains in a very early stage. Consequently, we herein investigate the therapeutic potential of two well-characterized SQS inhibitors (compounds **1, 2**, Scheme 1) in the treatment of lung cancer, aiming to disrupt tumor-associated cholesterol biosynthesis pathways. To this end, both **1** and **2** were initially screened for their cellular activity in various types of lung cancer cell lines, while their effect on cell cycle progression/proliferation, apoptotic/necrotic cell death, mitochondria membrane potential, as well as on migration/invasion was further evaluated. Lastly, the most active derivative was tested *in vivo* in an experimental animal model of NSCLC.

**Scheme 1:**
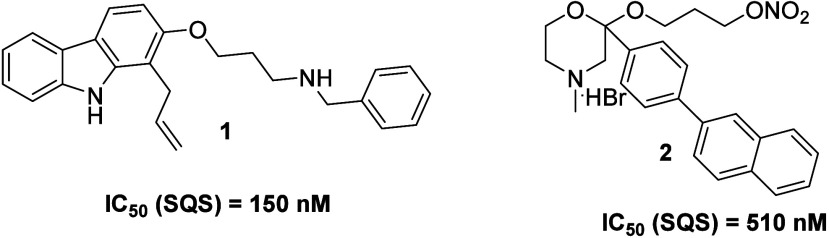
Structures of the Two Potent SQS Inhibitors Used in the Context of the Current Study^a^

a The inhibitory activity of each compound against SQS is also mentioned.

Given the lack of adequate experimental data for considering the inhibition of SQS as a novel and “druggable” therapeutic strategy to treat cancer, we initially assessed the impact of two potent SQS inhibitors (Figure 1) on lung cancer-related cell models, each one of them exhibiting different metastatic potential. Compound **1** belongs to the second generation of propylamine derivatives developed as orally active SQS inhibitors,^25^ while compound **2**, having been developed by our group, is an optimized morpholine SQS inhibitor (Scheme 1).^26^ The use of two chemically diverse SQS inhibitors in our assays was based on improving experimental consistency, identifying common characteristic pharmacological patterns of the compounds associated with SQS inhibition, validating findings, and accounting for potential off-target effects. Specifically, **1** and **2** were screened across (i) the highly metastatic mouse Lewis Lung Carcinoma (LLC) cell line expressing the ovalbumin (OVA) surface protein (LLC-OVA),^27^ (ii) the highly metastatic human NSCLC cell line H1299 derived from a lymph node metastasis,^28^ (iii) the human NSCLC cell line H358 derived from a bronchioalveolar carcinoma having the capability to produce high levels of the pro-inflammatory cytokines Interleukin-6 (IL-6), IL-8, and Growth-regulated α protein (GRO-α), which in turn can influence, depending on the context, the metastatic process,^29^ (iv) the widely used human alveolar basal epithelial A549 cell line derived from a lung carcinoma, which has a lower capacity to form metastatic lesions compared to the aforementioned cell lines,^30^ and (v) the mouse B16 melanoma cell line known for its ability to metastasize aggressively to the lung.^31^ In parallel, all derivatives were evaluated for their toxicity in synovial primary fibroblasts, selected as a normal cell model (negative control), to determine the compounds’ selectivity.

**Figure 1:**
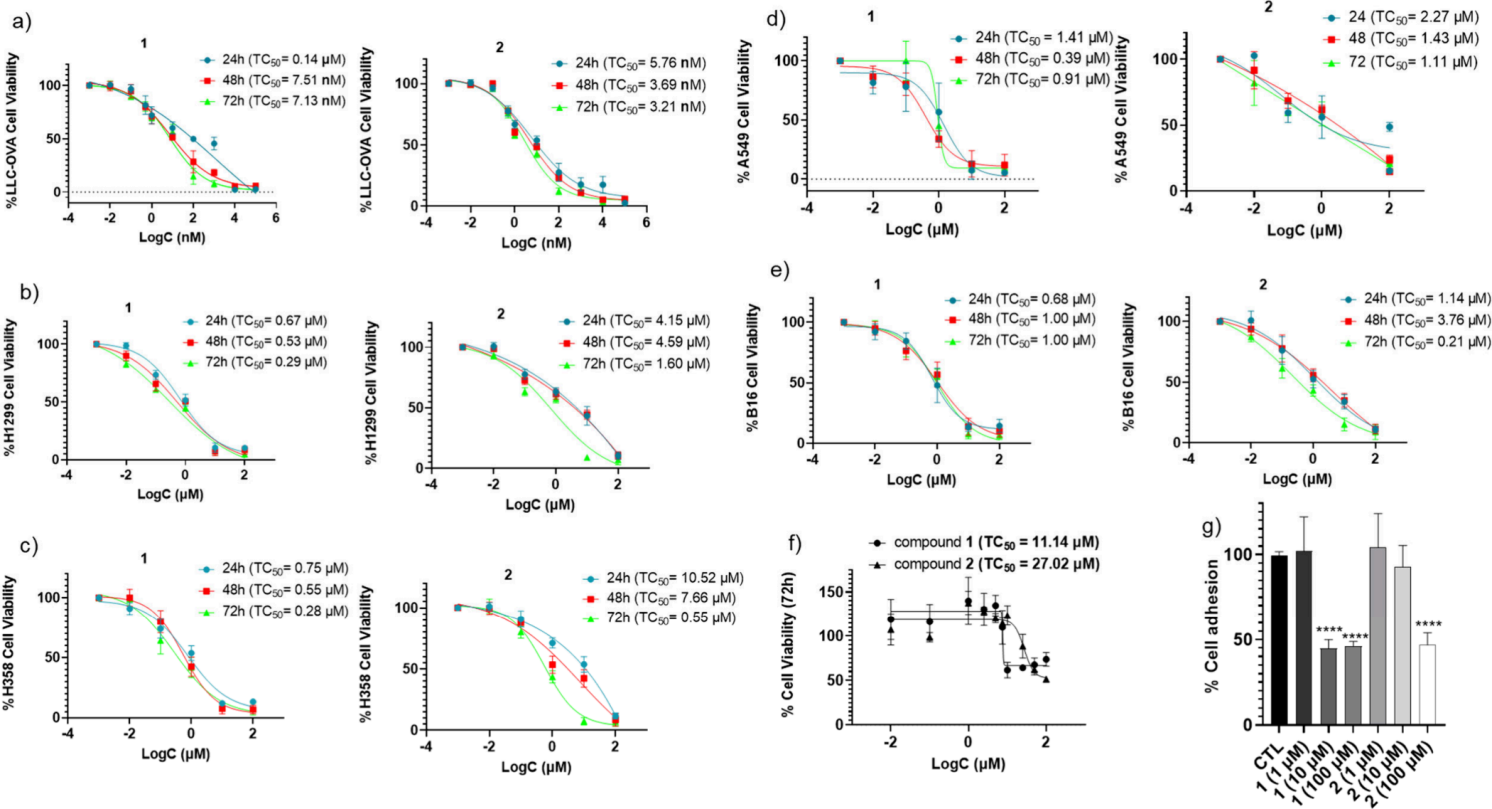
Effect of **1** and **2** on cell viability at 24, 48, and 72h in various mouse and human lung cancer cell lines displaying different metastatic potential, such as a) mouse LLC-OVA, b) human H1299, c) human H358, d) human A549, e) mouse B16, and f) normal mouse primary synovial fibroblasts after 72h of incubation. TC_50_ values are mentioned in parentheses. g) % decrease of cell adhesion by different concentrations of the compounds. TC_50_ value for each compound is the mean of 5 technical replicates. **** *P* < 0.0001.

The two SQS inhibitors were evaluated *in vitro* for their anticancer activity using the MTT viability assay; % levels of viable cells at 24, 48, and 72 h post treatment were measured, and the toxicity concentration (TC_50_) values of **1** and **2** for each time point were calculated (Figure 1). As displayed in Figure 1a, both analogues exhibited a powerful effect in LLC-OVA cells even at 24h, with their TC_50_ values ranging in the low nanomolar range. Furthermore, compounds **1** and **2** inhibited potently, and in a time-dependent manner, the cell growth in both human NSCLC cell lines tested, H1299 (Figure 1b) and H358 (Figure 1c), while they displayed at the same time a significant, albeit lower compared to LLC-OVA, effect in the A549 cell line (Figure 1d). Notably, they were also able to decrease remarkably the viability of the highly aggressive and metastatic lung B16 melanoma cells (Figure 1e), therefore confirming their pharmacologic potential across a variety of lung cancer cell lines. On the contrary, the toxicity caused by the two analogues in normal primary synovial fibroblasts was found to be much lower compared to that in cancer cell lines, with **1** and **2** only affecting significantly primary fibroblast viability at 72h and at much higher concentrations (Figure 1f). Of note, the cytotoxicity in fibroblasts/cytotoxicity in cancer cell lines ratio for **1** and **2** was found to be considerably high (up to 1,560 and 3,470 for **1** and **2**, respectively), an indication of high selectivity toward cancer cell lines and of a wide therapeutic window.

Given the potent anticancer effect of **1** and **2**, we then proceeded to study the mechanism through which the two analogues exert their cellular activity, examining whether they are involved, and to what extent, in affecting some of the most important (cancer) cellular processes, such as cell adhesion, cell cycle, and cell death progression, as well as cell migration/invasion. As depicted in Figure 1g, both compounds affected significantly, although at higher concentrations, cell adhesion, a process essential for both cell growth and survival, as well as for the communication and interaction among cells. Accordingly, a large proportion of cells is unable to adhere to the culture medium in the presence of compounds, resulting in the cells ultimately being driven to cell apoptosis.

Subsequently, the effect of **1** and **2** on the different phases of the cell cycle progression was assessed by Propidium Iodide (PI) cell viability flow cytometry analysis (Figure 2). To this end, the respective moderately and highly metastatic cancer cell lines A549 and LLC-OVA, respectively, were used, while, in order to acquire reliable results, **1** and **2** were tested in such concentrations (based on the MTT assay, Figure 1a,d) that allow the compounds to exert a descent pharmacological effect without at the same time affecting significantly cell viability and metabolic activity (toxicity <50%). In A549 cells (Figure 2a,b), both compounds acted exactly in the same way, reducing significantly the percentage of cells in the S (i.e., the proliferation phase) and G2 (i.e., premitotic phase) phases, while promoting an accumulation of cells in the G0/G1 phase (i.e., quiescent phase), indicating a G0/G1 arrest. In contrast, in LLC-OVA cells, flow cytometry analysis following treatment revealed a clear tendency of compound **1** to decrease the percentage of cells existing in the S phase and a pronounced accumulation of cells in the G2 phase, indicating that this compound induces a G2 cell cycle arrest (Figure 2c), with compound **2** showing an opposite trend, blocking DNA replication and progression through the cycle (Figure 2d). Therefore, it could be concluded that the inhibition of cell proliferation by the compounds, as shown by the MTT assays (Figure 1), could be attributed in part to the induction of premature cell cycle arrest, which could ultimately lead to apoptosis.

**Figure 2:**
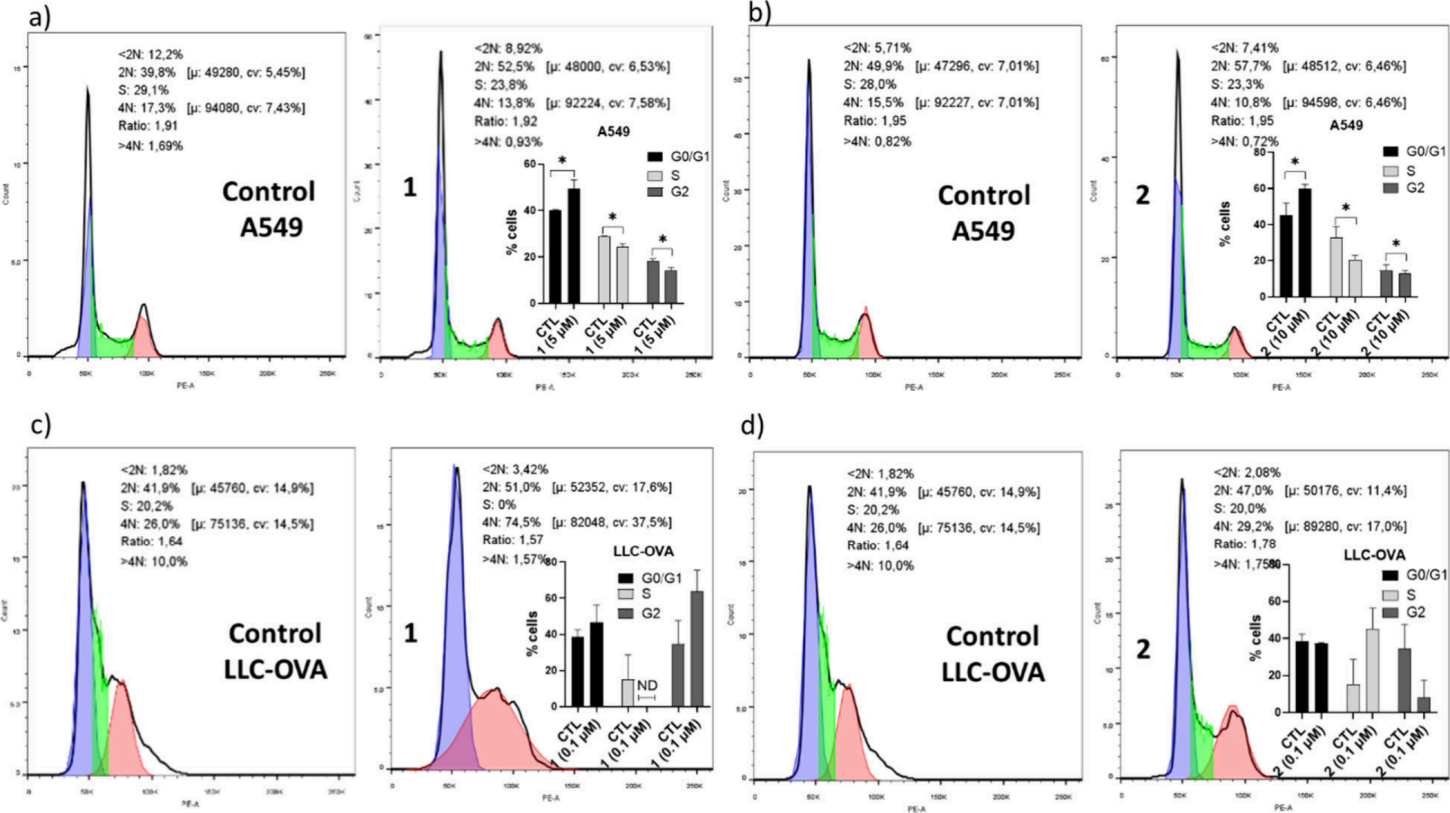
Effect of **1** and **2** on cell cycle progression: Flow cytometry imaging (G0/G1 phase is highlighted in blue, S phase in green, and G2 phase in purple) and measurement of % cells (subfigures) existing in G0/G1, S, and G2 phases upon incubation of A549 cells with compound **1** (a) and **2** (b) for 24h, at 5 and 10 μΜ concentration, respectively, as well as incubation of LLC-OVA cells with **1** (c) and **2** (d) at the concentration of 0.1 μΜ, in comparison to the respective controls (CTL, without compound treatment). Each sample was analyzed in three technical replicates. * *P* < 0.05, ND not detected.

To provide a deeper insight into this, the percentages of cells that are healthy, as well as those found in early apoptosis and late apoptosis/necrosis phases, were measured, with the purpose of figuring out whether and to what extent the compounds, and consequently the inhibition of SQS, are involved in cell death stages. The latter was performed with the Annexin V cell death assay, followed by flow cytometry analysis (FITC Annexin V). In A549 cells, both compounds did not show any statistically significant effect on the percentages of cells in the various phases of cell apoptosis/necrosis (data not shown). However, in LLC-OVA cells that are characterized by a very high metastatic capacity, **1** and **2** (0.1 μΜ) acted exactly in the same way, exhibiting a significant increase of the percentage of cells found in early apoptosis (Figure 3a,b). These findings indicate that SQS inhibition in LLC-OVA cells primarily induces a reversible early stage of programmed cell death. The occurrence of such apoptotic features may be related to metabolic collapse or overwhelmed membrane repair mechanisms due to disrupted lipid biosynthesis. Overall, the Annexin V assay outcomes support the proapoptotic effects of SQS inhibition and the subsequent induction of cell death, highlighting the potential of this enzyme as a metabolic vulnerability in highly metastatic lung carcinoma cells.

**Figure 3:**
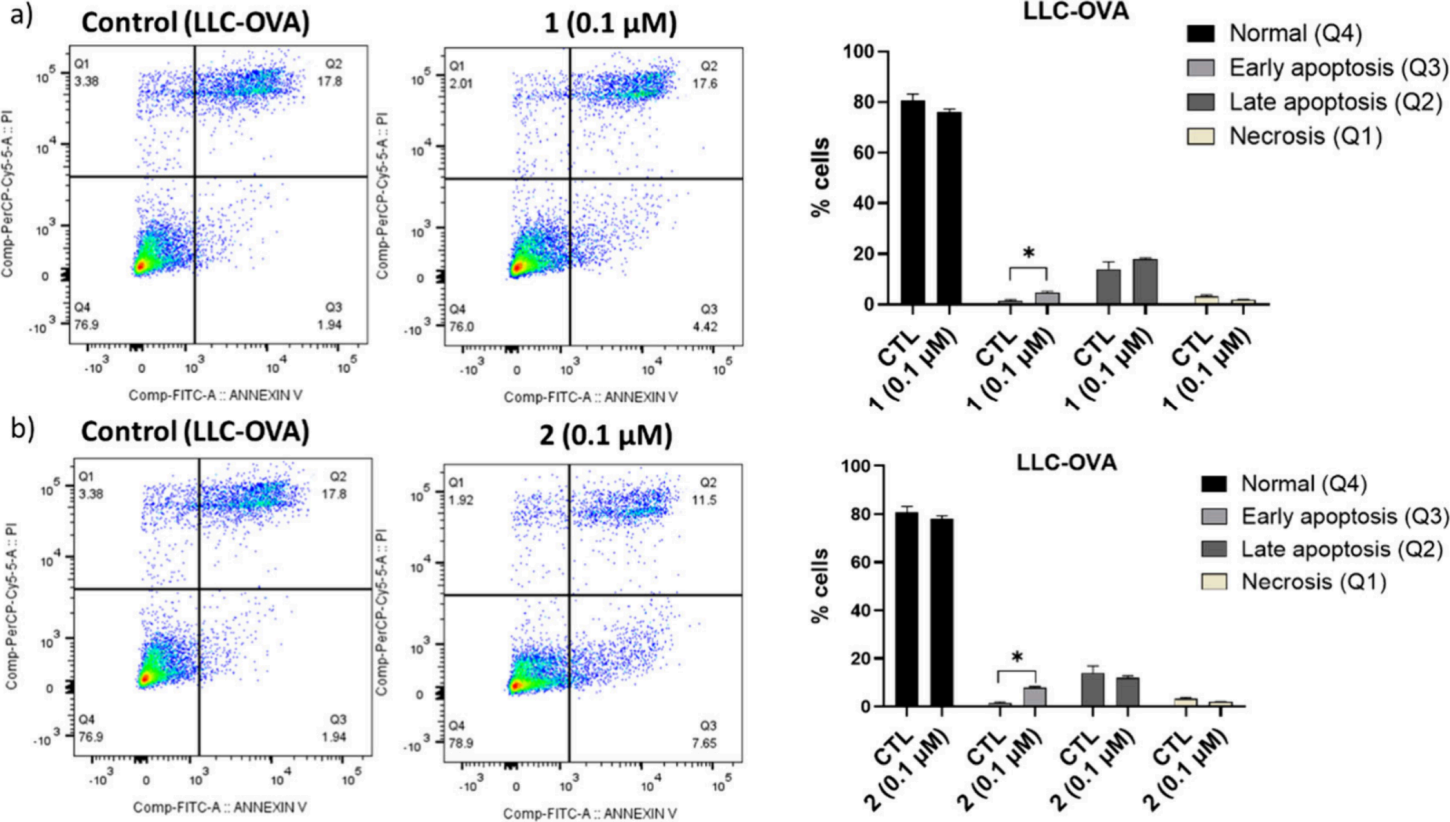
Effect of **1** (a) and **2** (b) on different phases of cell death. Flow cytometry imaging as well as percentages (%) of LLC-OVA cells existing in healthy (Q4), early apoptosis (Q3), late apoptosis (Q2), or necrosis (Q1), without compounds (Control, CTL) and upon treatment with compounds (0.1 μΜ) for 24h. Each sample was analyzed in three technical replicates. * *P* < 0.05.

Subsequently, we examined the effect of the compounds on mitochondria polarization using JC-10, a fluorescence dye forming either aggregates (red fluorescence) or monomers (green fluorescence) under different mitochondria conditions, thus acting as an indicator of mitochondria membrane potential (ΔΨm). As depicted in Figure 4, both derivatives increased dose-dependently the JC-10 aggregate/monomer ratio, thus leading mitochondria to hyperpolarization. The latter, which usually precedes the irreversible events of cell death (release of cytochrome c and caspase activation), can increase ROS production, trigger mitochondria permeability transition, disrupt calcium homeostasis, and cause ATP depletion, therefore predisposing cells to apoptosis/necrosis.^32^ This is particularly relevant in statin treatment where cholesterol synthesis inhibition usually causes mitochondrial hyperpolarization and oxidative effects in certain cells.^33^

**Figure 4:**
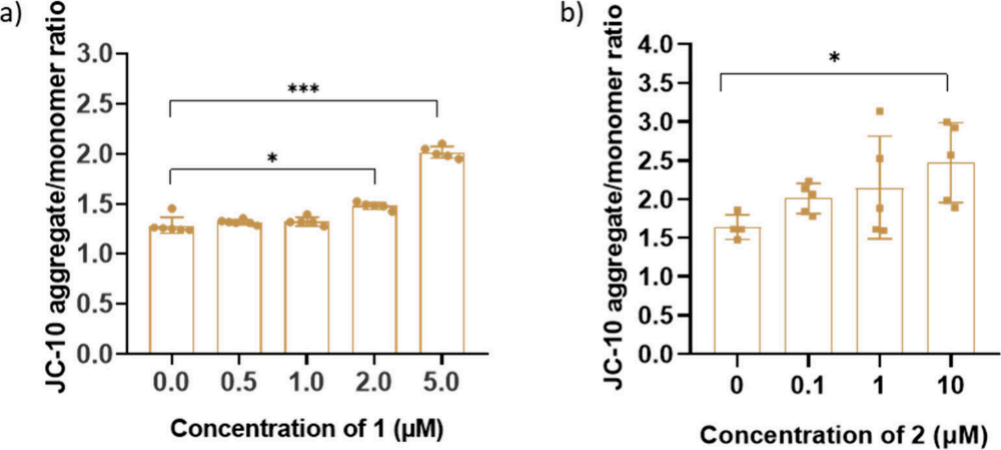
Mitochondrial membrane potential evaluated by JC-10 dye. The ratio of JC-10 aggregate/monomer fluorescence was measured in LLC-OVA cells treated with various concentrations of **1** (a) and **2** (b). * *P* < 0.05, *** *P* < 0.001.

Lastly, we investigated whether compounds **1** and **2** are capable of inhibiting cellular migration and metastasis, using LLC-OVA cells known to be endowed with a highly invasive/aggressive potential. Accordingly, two relevant to metastatic potential assays were used which measure different cellular behaviors, the wound healing (scratch) and the Boyden chamber (transwell) assays. The first one assesses 2D lateral migration without however distinguishing between migration and proliferation,^34^ while the second one quantifies mainly directional migration or invasion using a chemotactic gradient.^35^ As displayed in Figures 5a and 5b, treatment with both derivatives at very low concentrations (0.1 μΜ) led to a marked and statistically significant inhibition of lateral migration compared to untreated controls (CTL), reducing wound closure after 24h by approximately 45% (compound **1**) and 23% (compound **2**), respectively, indicating a strong suppressive effect on collective cell motility. The latter suggests that both SQS inhibitors may interfere with cytoskeletal dynamics or signaling pathways involved in migratory processes.^36^ Furthermore, in the Boyden Chamber assay (Figure 5c) performed using transwell inserts coated with Matrigel to mimic the extracellular matrix (ECM), stimulation with 10% FBS induced a robust invasion of the untreated LLC-OVA cells through the membrane after 24h. In contrast, cells pretreated with **1** and **2** at a very low concentration (0.1 μΜ) exhibited 30% and 25% reduction in invasive capacity, respectively, an indication of an impaired chemotactic response due to the reduced ability of cells to degrade and traverse ECM-like barriers.

**Figure 5:**
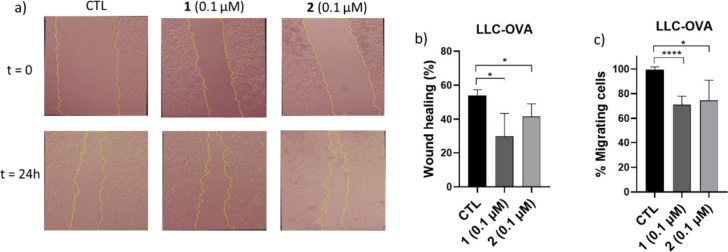
Effect of **1** and **2** on cell migration/metastasis: a) Representative photos of wound healing (*t* = 0 and 24h) of control (CTL, without treatment) LLC-OVA cells, and upon treatment with **1** and **2** at 0.1 μΜ. b) Quantification (%) of wound closure (displayed in Figure a) after 24h of incubation. c) Percentage (%) of migrating/invading LLC-OVA cells from the upper to the lower compartment separated by a porous membrane (Boyden chamber assay) without (CTL) and upon treatment with **1** and **2** at 0.1 μΜ for 24h. Each sample was analyzed in 4 technical replicates. * *P* < 0.05, **** *P* < 0.0001.

After confirming the cell-based anticancer potential of both SQS inhibitors in different lung cancer cell lines, as well as investigating their impact on cell cycle progression, the different phases of cell death, mitochondria membrane potential and metastasis, we then conducted an *in vivo* experiment, using a metastatic mouse model, to evaluate the capability of SQS inhibitor **1** to inhibit the high propensity of LLC-OVA cells for distant spread/colonization, especially in the lung. The selection of **1** over compound **2** for testing in the *in vivo* study was relied on: (i) the more potent pharmacological effect it exhibited in the cell-based assays performed (Figures 1–5), (ii) the fact that it has displayed a potent antihypercholesterolemic/antihyperlipidemic activity *in vivo*,^25^ and (iii) the lower intrinsic clearance rate and longer half-life time it demonstrated in the *ex vivo* metabolic stability studies performed using mouse liver microsomes/NADPH (Table S1). Accordingly, 5 × 10^5^ LLC-OVA cells dispersed in 100 μL of DMEM were injected intravenously (*iv*) via the tail vain in each mouse (C57BL/6), thus providing them with the opportunity to lodge in the lung capillaries, where they extravasate and form metastatic nodules in the lung parenchyma. Metastatic growth is typically visible within 10–21 days, depending on cell dose and immune status. Compound administration was performed intraperitoneally (*ip*), both at a preventive (PTR) and a therapeutic treatment regimen (TTR). In PTR, treatment with **1** (*ip*, 15 mg/kg, twice per day) began on day 0, when *iv* LLC-OVA loading took place, and continued until day 21 (end of the study), while in the TTR, the compound was administered from day 10, when the metastatic nodules in the lung become visible, to day 21, using the same dose scheme as in PTR.

Intravenous injection of LLC-OVA gave rise to the formation of severe lung vascular leak and pulmonary edema after 21 days, as indicated by the increase in the inflammatory cells in the BALF (Figure 6a, compare Normal to CTL group). Furthermore, the tumor burden in the lungs was significantly increased (21st day), characterized by extensive metastatic nodules visible upon gross inspection. Histological analysis (H&E staining) confirmed widespread tumor infiltration, with loss of normal alveolar architecture and dense tumor cell accumulation throughout the lung parenchyma (Figure 6b). Quantitative analysis revealed a progressive increase in tumor area, with approximately a 15–30% lung involvement in the majority of animals (Figure 6c, compare Normal to CTL group). Variations in tumor growth are expected in this model ascribed to differences in cancer cell lung colonization and proliferation among mice. On the contrary, compound **1** markedly reduced vascular permeability and inflammation with either treatment regimen, restoring them to normal levels (Figure 6a). Notably, in the PTR, **1** exhibited a remarkable reduction in the number and size of metastatic nodules compared to the control group (Figure 6b), while quantitative analysis showed a 60% decrease in tumor burden (Figure 6c). Lastly, in the TTR, a tendency of **1** to inhibit the lung tumor burden was also observed (27% reduction, Figure 6b,c), although not reaching statistical significance. Of note, zaragozic acid A, the only SQS inhibitor tested previously *in vivo* in a similar lung cancer metastasis model, was administered *iv* and only at a preventive mode.^8^

**Figure 6:**
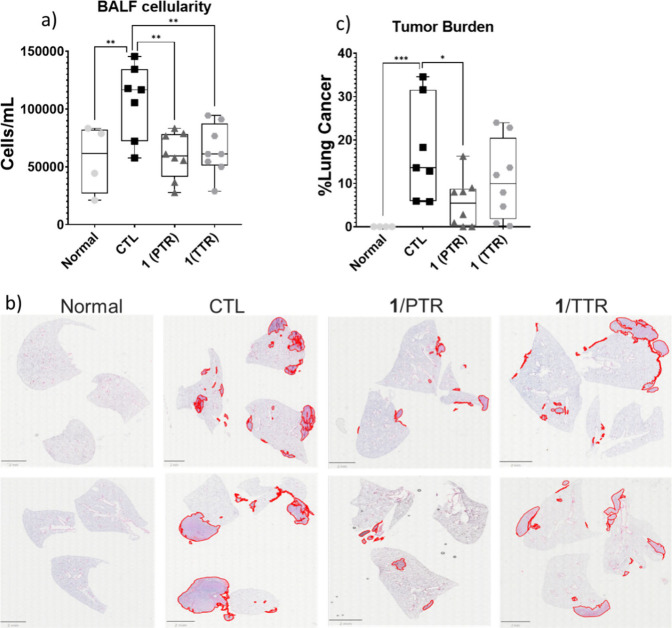
*In vivo* effect of **1** in the LLC-OVA experimental metastatic mouse model. a) Inflammatory cell numbers in Bronchoalveolar Lavage Fluids (BALFs), as counted with a hematocytometer. b) Representative images of lung sections (4 μm thick) from mice of the indicated treatment groups (Normal, CTL, PTR, and TTR), stained with Hematoxylin and Eosin (H&E). For better clarity, the cancer nodules have been marked. c) Quantification tumor burden, calculating the percentage of surface area covered by cancer in relation to the entire surface of each lung, from each section and sample. * *P* < 0.05, ** *P* < 0.01.

Most studies in literature report that the overexpression of SQS is significantly associated with poor prognosis in patients suffering from lung cancer, whereas the genetic downregulation/silencing of SQS is accompanied by a potent anticancer effect both in cells and *in vivo*.^8^ Nevertheless, the evaluation of SQS inhibitors as anticancer agents is rudimentary thus far and limited only to two compounds, the natural product zaragozic acid A^8^ (tested *in vivo* in a different mouse lung cancer metastasis model than that of the current study) and the synthetic quinuclidine derivative YM-53601 (tested only in glioblastoma cell-based assays).^12^ In the present work, we attempted to provide clear evidence about the potential of this enzyme as a “druggable” target in lung cancer by using in the same study two chemically diverse SQS inhibitors (compounds **1** and **2**) and testing them not only in mouse but also in human lung cancer-related cell lines, aiming at assessing in part the human translational potential of SQS inhibition by small molecules. In addition, we examined the impact these derivatives have on cell adhesion, cell cycle progression, mitochondria membrane potential, and the different phases of cell death, thus offering some initial groundwork for the mechanistic underpinning of SQS inhibition in cancer cells, but at the same time underscoring the need for further investigation. Lastly, *in vivo* studies corroborated the potent effect of SQS inhibition, especially at a preventive level.

In conclusion, SQS is probably a viable and promising drug target for the fight against malignant cancer such as lung cancer. However, further studies are necessary in order to clarify the optimal conditions and molecular mechanisms induced by SQS inhibition in the biological system [e.g., caspase cleavage, B-cell lymphoma 2 (Bcl-2) family proteins, gene expression changes, reactive oxygen species (ROS) measurements], as well as to explore SQS’s regulatory mechanisms and its role in lung tumor biology. Moreover, further development of new series of SQS inhibitors together with optimization of compound specificity, delivery systems, or administration routes (e.g., through inhalation), and combination strategies with other therapies [e.g., statins, mammalian target of rapamycin (mTOR) inhibitors, immune checkpoint inhibitors] will exploit fully the prospects of this enzyme in combating both primary tumors and metastatic disease, lending at the same time special support for its translational promise.

Safety statement: No unexpected or unusually high safety hazards were encountered.

## Supplementary Material



## Notes

Experimental procedures, ^1^H NMR spectra, supporting table, and references (PDF)

## Abbreviations

SQS: squalene synthase
FPP: farnesyl pyrophosphate
EGFR: epidermal growth factor receptor
PI3K: phosphoinositide 3-kinase
Ras: rat sarcoma
MAPK: mitogen-activated protein kinase
NSCLC: non-small cell lung cancer
ERK: extracellular-signal-regulated kinase
NF-kB: nuclear factor kappa-light-chain-enhancer of activated B cells
MMP1: matrix metallopeptidase 1
TNFR1: tumor necrosis factor receptor 1
LLC: Lewis Lung Carcinoma
OVA: ovalbumin
IL-6: Interleukin-6
IL-8: Interleukin-8
GRO-α: growth-regulated α protein
PI: propidium iodide
ECM: extracellular matrix
*iv*: intravenously
*ip*: intraperitoneally
BALF: bronchoalveolar lavage fluid
PTR: preventive treatment regimen
TTR: therapeutic treatment regimen
mTOR: mammalian target of rapamycin
Bcl-2: B-cell lymphoma 2
ROS: reactive oxygen species
