# Supplementary material for: Unraveling the Potential Pathogenic Role of Squalene Synthase (SQS) in Lung Cancer Using Enzyme Inhibitors as Molecular Tools
Source: ACS Med Chem Lett. 2025 Sep 11;16(10):2041–8. doi: 10.1021/acsmedchemlett.5c00458 (PMC12516386; doi:10.1021/acsmedchemlett.5c00458)
Supplement: Supplementary file 1 [file ml5c00458_si_001.pdf]

Unraveling the Potential Pathogenic Role of Squalene Synthase (SQS) in Lung Cancer Using Enzyme Inhibitors as Molecular Tools.

Theodora Katavati,[1,3#] Filippos P. Chatzipieris,[1,3#] Christiana Magkrioti,[2] Elli-Anna Stylianaki,[2] Emmanouil Aerakis,[1] Sofia Grammenoudi,[2] Maria Tsoumakidou,[1] Vassilis Aidinis,[2] Alexios N. Matralis,[1*] Angeliki P. Kourounakis[3*]

[1]Institute for Bioinnovation and [2]Institute for Fundamental Biomedical Research, Biomedical Sciences Research Center "Alexander Fleming", 16672 Athens, Greece. [3]Department of Medicinal Chemistry, School of Pharmacy, University of Athens, 15771 Athens, Greece.

# these authors contributed equally to this work.

** corresponding authors: E-mails: matralis@fleming.gr, angeliki@pharm.uoa.gr*

Supplemental Information

Table of contents

1.Experimental Section……………………………………………………………………………2

1.1 Synthesis……………………………………………………………………………..………2

1.2 MTT cytotoxicity assay in cancer and normal cell lines……………………………..…………..2

1.3 Cell adhesion assay………………………………………………………………………..2

1.4 Cell cycle progression – Propidium Iodide (PI) cell viability flow cytometry assay………………..2

1.5 Cell death assay (FITC-Annexin V)…………………………………………………………3

1.6 JC10 mitochondrial membrane potential assay……………………………………………...…3

1.7 Wound healing assay………………………………………………………………………...3

1.8 Boyden chamber assay……………………………………………………………….…………3

1.9 Software used for cell-based assays………………………………………………………….4

1.10 *Ex vivo* metabolic stability assay…………………………………………………….………..4

1.11 Mice living conditions and performance of experimental study………………….……..…….4

1.12 Cancer induction………………………………………………………...……………….5

1.13 BALF collection…………………………………………………………………….…………5

1.14 Tissue isolation…………………………………………………………………..……………5

1.15 Tissue processing……………………………………………………………………………...5

1.16 Deparaffinization and H&E (Hematoxylin and Eosin) Staining of Sections…………………..…...5

1.17 Analysis……………………………………………………………………………………….6

2. Supporting Table S1……………………………………………………………………………6

3. ^{1}H-NMR spectra……………………………………………………………………………...7

4. References……………………………………………………………………………………….9

1. Experimental Section

1.1 Synthesis. Compounds **1** and **2** were synthesized according to the synthetic procedures described elsewhere and characterized by ^{1}H-NMR and MS.[1,2] Both compounds are ≥95% pure by HPLC.

1.2 MTT cytotoxicity assay in cancer and normal cell lines. 5,000 cells were seeded in 96-well plate and were incubated for 6-8 hours. Then, the medium was removed, and wells were washed with PBS. Fresh culture medium, containing, instead of 10%v/v FBS, 10%v/v BSA 0.2% (starvation medium) for starvation conditions, was added and the well-plate was incubated for 16 hours. Afterwards, wells were filled with fresh starvation medium which contained the corresponding inhibitor solution. The well-plate was incubated for 24, 48 or 72 hours, depending on the study. The supernatant was then removed from each well, which was washed with medium in the absence of phenol red dye (DMEM plenol red free). In the end, an appropriate volume of MTT solution (5 mg/mL DMEM phenol red free) was added per well and the plate was incubated for 4 hours to form purple crystals. The supernatant was removed, DMSO was added, and wells were kept in the dark for 30 minutes with simultaneous stirring. Absorption was measured by photometry at a wavelength of 570nm and reference 690nm.

1.3 Cell adhesion assay. 10,000 cells were seeded in a 96-well plate and at the same time test compounds were added in each well, following incubation for 5 hours at 37°C, 95% humidity and 5% CO_2. Then, 4% w/v paraformaldehyde (Paraformaldehyde, PFA) was added to adhere the cells to the plate, and the plate was left for 10 minutes at room temperature. PFA was removed, wells were washed with PBS in order to remove detached cells, and crystal violet was added and left for 10 minutes at room temperature in the dark. The well-plate was washed under running water and dried well. 2% sodium dodecyl sulfate (SDS) was subsequently added, and left for 30 minutes at room temperature in the dark under stirring. Absorption was measured by photometry at a wavelength of 550 nm.

1.4 Cell cycle progression – Propidium Iodide (PI) cell viability flow cytometry assay. 300,000 cells were seeded in a well-plate for 24 hours at 37°C, 95% humidity and 5% CO_2, and grown to approximately 90% confluency. Then, the cell medium was removed, wells were washed with PBS, fresh complete cell medium was added followed by inhibitor treatment. After incubation (at 37°C, 95% humidity and 5% CO_2) for 24 hours, the cells were washed with PBS, followed by PBS refill of each well. Cells were detached from each well using a scraper, transferring the suspension to flow cytometry tubes. Following centrifugation of the tubes at 350 RCF/5 minutes/4 ^{o}C, and decantation of the supernatant, a mixture of ice-cold-70% ethanol was added while stirring, followed by resting at room temperature for 30 minutes. The sediment was suspended in PBS, centrifuged at 700 RCF/ 5 minutes/4 ^{o}C, and the supernatant was removed. Each tube was treated with ribonuclease (RNase, 100 μg/mL), and the suspension was transferred to Eppendorf vials. After standing for 30 minutes at 37 ^{o}C, a PI solution (50 μg/mL) was added, and the suspension was transferred to new flow cytometry tubes. Upon standing in the dark for 30 minutes at room temperature, contents of each tube were analyzed by flow cytometry.[3]

1.5 Cell death assay (FITC-Annexin V). Cells (150,000) were seeded in a 6-well plate and incubated for 24 hours at 37°C, 95% humidity and 5% CO_2. After removing the medium, washing with PBS, and refilling with fresh complete medium, the SQS inhibitor was added at the indicated concentration. The well-plate was re-incubated for 24 hours, the culture medium was removed, and the cells were washed with PBS. In each well, 1x Trypsin-EDTA was added, and left for 15 min at room temperature. Culture medium was added to the plate, which was immediately aspirated to remove any particles. Then, cells were collected in flow cytometry tubes, centrifuged (350 RCF/5 min/9 ^oC), the supernatant was removed, and the cells were re-washed with PBS and re-centrifuged. The supernatant was decanted in new tubes, followed by addition of Binding Buffer solution (0.14 M NaCl, 10 mM Hepes pH=7.4, 2.5 mM $CaCl_2$) and Annexin V. Tubes were left in the dark for 20 min, then PI (1 mg/mL) was added and left again in the dark for another 20 min before analyzed by flow cytometry.[4]

1.6 JC10 mitochondrial membrane potential assay. 5,000 cells were seeded in a 96-well plate and were incubated for 6-8 hours. Then, the medium was removed, and wells were washed with PBS. Fresh culture medium, containing, instead of 10%v/v FBS, 10%v/v BSA 0.2% (starvation medium) for starvation conditions, was added and the well-plate was incubated for 16 hours. Afterwards, wells were filled with fresh starvation medium which contained different concentrations of inhibitor solution. The well-plate was incubated for 24 hours. The supernatant was then removed from each well in the dark, and JC10 solution (final concentration 5 μM) in acetone was added per well. After then, the well-plate was incubated for 15 minutes at 37 ^oC. The supernatant was removed from each well and PBS was added per cell in the dark. At the end, fluorescence was measured (Tecan Infinite M200 Pro plate reader) at excitation/emission wavelengths: 535nm/595 nm for JC-10 polymers and 485 nm/535 nm for JC-10 monomers.

1.7 Wound healing assay. Cells were cultured in 24-well plates for 24 hours at 37°C, 95% humidity and 5% CO_2, and grown to approximately 90% confluency (seeded at approximately 75,000 cells per well). Then, the cell monolayer was scratched in a straight line using a pipette tip, washed gently, to remove detached cells and then wells were refilled with fresh medium and treated with the test compounds. Photographs were taken with an inverted phase microscope at t = 0. Then, well plates were incubated at 37 ^oC, 95% humidity, 5% CO_2 for 24 hours and photographs were taken at t = 24 hours. The images were finally processed in order to measure the wound area.[5]

1.8 Boyden chamber assay. Cells were seeded (150,000 cells per well) in 6-well plate for 24 hours at 37°C, 95% humidity and 5% CO_2, and grow to approximately 90% confluency in appropriate medium. After washing with PBS, trypsin was added, incubated for a short period of time, followed by addition of culture medium. The content of each well was centrifuged (300 RCF/5 minutes/4 ^oC), cells were seeded (100,000 per well) on the upper part of a 24-well plate with a semi-permeable membrane (transwell chamber), inhibitor was added at the indicated concentration, and the whole system was incubated for 24 hours at 37 ^oC. Before seeding, the bottom of the plate was coated with fibronectin (FN), incubated for 2 hours and culture medium, enriched with 10%v/v BSA, was added in the bottom part. Afterwards, the filter with the semi-permeable membrane and the medium were removed and the surface of the

top was cleaned for removing non-migrating cells. Crystal violet was added to new wells in which the filters were placed and left for 5 minutes at room temperature, washed with water and left for another 30 minutes to dry. Acetic acid was added in new wells, in which filters were placed and left for 15 minutes at room temperature under stirring. Absorption of the solution at a wavelength of 570 nm was finally measured by photometry.[6]

1.9 Software used for cell-based assays. The emission data were plotted in GraphPad Prism 8.0. For the PI cell viability flow cytometry analysis and cell death assay (FITC-Annexin V), data analysis were made using the FlowJo VX 10.0.7r2 Analysis Software. Image J 1.50b Launcher Symmetry Software was used to measure the area of the wound at each time point in the wound healing assay.

1.10 *Ex vivo* metabolic stability assay. The stability of compounds **1** and **2** against microsomal degradation was studied using male mouse liver microsomes (C57BL/6). The test compound (final concentration 0.1 μM) was incubated with liver microsomes (0.5 mg/mL) in 100 mM potassium phosphate buffer (pH 7.4) containing 1mM NADPH for 0, 15, 30, or 60 min in a total volume of 100 μL. A reaction mixture containing heat-inactivated microsomes (95 °C, 5 min) was prepared as a control. Propranolol was used as a positive control. At the end of the respective incubation period, microsomal activity was terminated by addition of 500 μL of MeCN and subsequent centrifugation at 1700 g for 5 min. The concentration of the compounds in the supernatant at each time point was determined by LCMS-ESI. Metabolic stability, expressed as percent of the parent compound remaining, was calculated by comparing the peak area of the compound at the time point relative to that at time-0. The half-life ($t_{1/2}$) was estimated from the slope of the initial linear range of the logarithmic curve of compound remaining (%) vs. time, assuming the first-order kinetics. The apparent intrinsic clearance (CL_{int}, in μL/min/mg) was calculated according to the following formula: $CL_{int} = 0.693/(t_{1/2}$ x mg protein/μL).[7]

1.11 Mice living conditions and performance of experimental study. The care of the experimental animals, as well as the experimental routes followed were carried out at the Biomedical Sciences Research Center “Alexander Fleming”. Male C57BL/6 mice were used as experimental animals. Experimental animals were divided into homogeneous groups in terms of sex, age and weight and were kept in suitable cages in an air-conditioned room (21 – 24 ^{o}C), humidity 50 – 60%, with ventilation (10 – 12 air changes per hour). The day/night cycle was 12 hours light (7:00 – 19:00) and 12 hours dark. The mice had free access to water and chow. The water was provided by the local city supply network “EYDAP” and was irradiated by a UV (Ultra Violet) lamp in order to disinfect it. Chow pellets were provided by the Italian Company Mucedola and contained 19% protein and 5% fat. Bedding was sterilized by autoclaving, and it contained only minimal amounts of dust (non-allergic). All procedures received approval from the Protocol Evaluation Committee (PEC) of the Biomedical Sciences Research Center “Alexander Fleming” and were licensed by the Veterinary Authority of the Attica region, Greece (#700105, 2024). The institution’s Animal Welfare Body (AWB) provided oversight of animal welfare compliance.

1.12 Cancer induction. Lewis Lung Carcinoma cell line (LLC) was obtained from the American Type Collection Cultures (Manassas, VA) transduced with OVAmCherry lentiviruses and sorted based on fluorescent protein expression. Cancer cells were maintained in DMEM, containing 10% heat-inactivated FBS, 1% L-glutamine, and 1% penicillin/streptomycin (Gibco). For the cancer model, cancer cells were thawed from frozen stocks and propagated in medium ($DMEM^+$ 10%, FBS^+ 0.1%, 2 Mercaptoethanol) for 5–7 days with one intervening passage *in vitro*. On the day of injection, cells were harvested by incubation in 0.05% trypsin-EDTA and washed three times with endotoxin-free PBS. For the metastatic model, mice were injected intravenously via the tail vein with 5 x 10^5 LLC cells in 100 μL DMEM. For the experiment, four groups were formed: a Normal group consisting of 4 mice which were not injected with LLC-OVA cells for tumor induction nor treated with **1**, a Control group (CTL) consisting of 7 mice that were injected with LLC-OVA cells for tumor induction but did not receive treatment with **1**, and two treatment groups (both injected with LLC-OVA cells), a PTR (preventive treatment regimen) group consisting of 8 mice receiving compound **1** for 21 days (from day 0 to day 21), and a TTR (therapeutic treatment regimen) group consisting of 8 mice receiving compound **1** for 11 days (from day 10 to day 21).[8]

1.13 BALF collection. The bronchoalveolar lavage fluid (BALF) was received by three lung fillings with saline and collection of each of them. The supernatant of the first wash of BALF (bronchoalveolar lavage) was collected for liquid biopsy (1200 rpm, 10 min). The cells that make up the sediment from all three washes were also collected, pooled into one sample and counted.

1.14 Tissue Isolation. From the total 5 lobes of the lung, 3 lobes (superior, inferior and post-caval lobes) were isolated for histology experiments, while the other two (the middle lobe and the left lung) were kept at -80°C.

1.15 Tissue Processing. 3 lobes of the lung (superior, inferior and post-caval lobes) were used for histology experiments; 1 mL of formalin was injected during sacrifice and the tissues were processed, then immersed in formalin (4°C), and after one day in PBS (4°C). Tissues were subsequently enclosed in cassettes, and filled with paraffin. Tissue sections were made (4 μm thick). Superficial sections were obtained at 20 μm and 40 μm, while deep sections were taken at depths of 250 μm and 500 μm. All sections were placed on proper slides.

1.16 Deparaffinization and H&E (Hematoxylin and Eosin) Staining of Sections. The slides with the superficial and deep sections were prepared for deparaffinization and H&E staining, as follows:

<u>a) Deparaffinization</u>

1. Immersion in xylene 20 times (20x) with insertion-extraction of the comb for 1 second (1”) each time, 2. Immersion in xylene for 20x 1”, 3. Immersion in ethanol 100° for 20x 1” and then at 96° for 20x 1”, at 75° for 20x 1” and at 50° for 20x 1” and 4. Immersion in deionized water until the next procedure (approximately 5 min).

<u>b) Staining</u>

1. Immersion in hematoxylin for 1x 3” for staining nuclei in tissue cells, 2. Immersion in deionized water and successive washes until the deionized water of the washes runs clear, 3. Immersion in Scott’s buffer for 5 minutes, 4. Immersion in acidified ethanol with 1% acetic acid, 5. Immersion in eosin for 10x 1”, for cytoplasmic staining of tissue cells, 6. Immersion in 50° ethanol for 10x 1” and then at 75° for 10x 1”, at 96° for 10x 1” and at 100° for 10x 1” and 7. Immersion in xylene for 10x 1”, 8. Immerse in a second xylene for 10x 1” and leave in it until the placement of the coverslip (dry the slide and use DPX oil). Finally the sections were photographed, and the photographs were analyzed with the QuPath - 0.5.1 program to calculate the percentage of surface area covered by cancer in relation to the entire surface of each lung section, from each sample.

1.17 Analysis. Data are expressed as the mean ± SD. Where indicated, statistical comparisons were made using Student’s t test, and a statistically significant difference was inferred if $P < 0.05$.

2. Supporting Table S1

Table S1. *In vitro* metabolic stability of compounds **1** and **2** using mouse liver microsomes. The intrinsic clearance (CL_{int}) and half-life time ($t_{1/2}$) values are shown.

Compound	Mouse Clearance (CL_{int}) (µL/min/mg)	$t_{1/2}$ (min)
1	346.9	20
2	1618.9	4.3
Propanolol	713.0	7.9

3. ^{1}H-NMR spectra

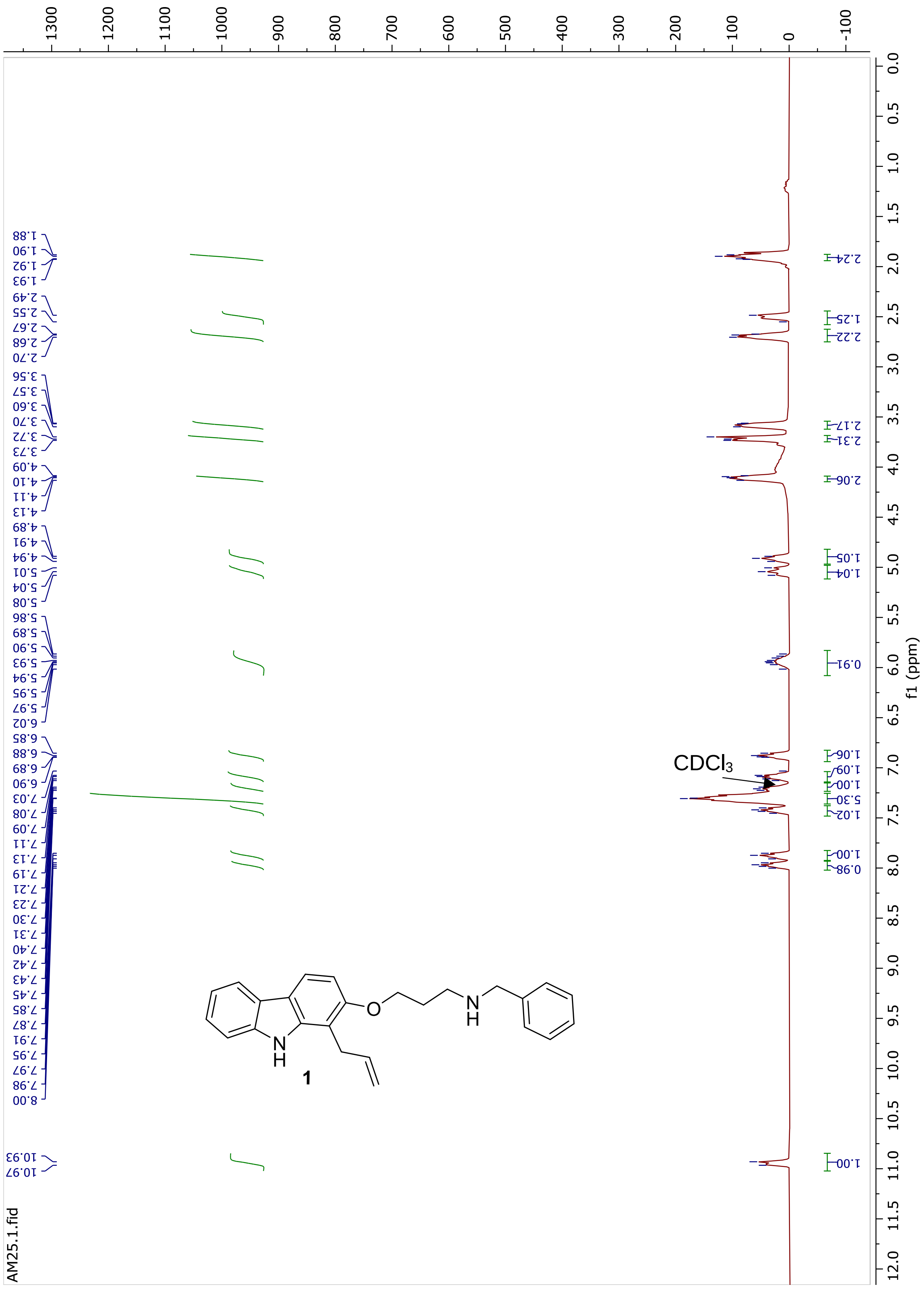

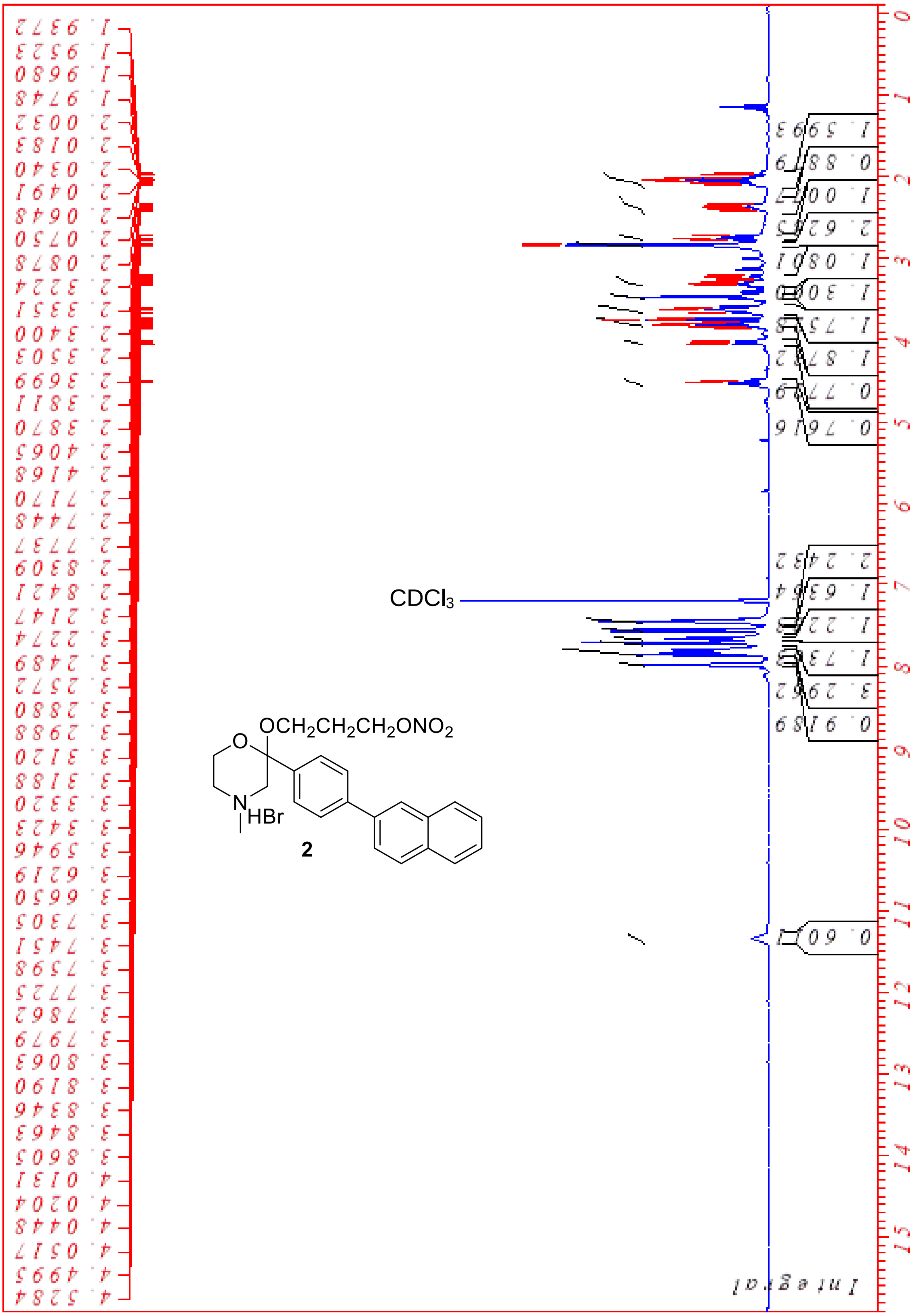
CDCl3
OCH2CH2CH2ONO2
O
N
HBr
2

4. References

1) Ishihara, T.; Kakuta, H.; Moritani, H.; Ugawa, T.; Yanagisawa, I. Synthesis and Biological Evaluation of Novel Propylamine Derivatives as Orally Active Squalene Synthase Inhibitors. *Bioorganic & Medicinal Chemistry* **2004**, *12* (22), 5899–5908. https://doi.org/10.1016/j.bmc.2004.08.033.

2) Matralis, A. N.; Kourounakis, A. P. Optimizing the Pharmacological Profile of New Bifunctional Antihyperlipidemic/Antioxidant Morpholine Derivatives. *ACS Med. Chem. Lett.* **2019**, *10* (1), 98–104. https://doi.org/10.1021/acsmedchemlett.8b00469.

3) Riccardi, C.; Nicoletti, I. Analysis of Apoptosis by Propidium Iodide Staining and Flow Cytometry. *Nat Protoc* **2006**, *1* (3), 1458–1461. https://doi.org/10.1038/nprot.2006.238.

4) Schutte, B.; Nuydens, R.; Geerts, H.; Ramaekers, F. Annexin V Binding Assay as a Tool to Measure Apoptosis in Differentiated Neuronal Cells. *Journal of Neuroscience Methods* **1998**, *86* (1), 63–69. https://doi.org/10.1016/S0165-0270(98)00147-2.

5) Rodriguez, L. G.; Wu, X.; Guan, J.-L. Wound-Healing Assay. In *Cell Migration*; Humana Press: New Jersey, 2004; Vol. 294, pp 023–030. https://doi.org/10.1385/1-59259-860-9:023.

6) Brown, K.; Sugrue, A.; Modi, K.; Light, R.; Conley, K.; Cox, A.; Bender, C.; Miles, S.; Valentovic, M.; Dasgupta, P. An Experimental Protocol for the Boyden Chamber Invasion Assay with Absorbance Readout. *BIO-PROTOCOL* **2024**, *14* (1350). https://doi.org/10.21769/BioProtoc.5040.

7) Obach, R. S.; Baxter, J. G.; Liston, T. E.; Silber, B. M.; Jones, B. C.; Maclntyre, F.; Rance, D. J.; Wastall, P. The prediction of human pharmacokinetic parameters from preclinical and in vitro metabolism data. *J. Pharmacol. Exp. Ther.* **1997**, *283* (1), 46-58.

8) Kerdidani, D.; Chouvardas, P.; Arjo, A. R.; Giopanou, I.; Ntaliarda, G.; Guo, Y. A.; Tsikitis, M.; Kazamias, G.; Potaris, K.; Stathopoulos, G. T.; Zakynthinos, S.; Kalomenidis, I.; Soumelis, V.; Kollias, G.; Tsoumakidou, M. Wnt1 Silences Chemokine Genes in Dendritic Cells and Induces Adaptive Immune Resistance in Lung Adenocarcinoma. *Nat Commun* **2019**, *10* (1), 1405. https://doi.org/10.1038/s41467-019-09370-z.
